# Blue Nevus Associated With Acquired Dermal Melanocytosis on the Back

**DOI:** 10.7759/cureus.65428

**Published:** 2024-07-26

**Authors:** Shin Iinuma, Takahiro Kobayashi, Yuto Fujiki

**Affiliations:** 1 Dermatology, Japanese Red Cross Kitami Hospital, Kitami, JPN; 2 Dermatology, Asahikawa Medical University, Asahikawa, JPN; 3 Pathology, Kitami Red Cross Hospital, Kitami, JPN

**Keywords:** pigmentary disease, melanocyte, dermal melanocytosis, blue nevus, acquired dermal melanocytosis

## Abstract

Dermal melanocytosis includes various congenital and acquired pigmentary disorders characterized by dermal dendritic melanocytes. Blue nevi typically present as papulonodular lesions, whereas other dermal melanocytoses manifest as patches. This report describes a case of a blue nevus associated with acquired dermal melanocytosis on the back of a 46-year-old Japanese woman. The patient presented with a black nodule on a blue-greyish hyperpigmented area on the upper back. Histopathological analysis of the nodule confirmed a common blue nevus, whereas the adjacent hyperpigmented area showed features consistent with acquired dermal melanocytosis. Blue nevi and acquired dermal melanocytoses share a common pathophysiology involving ectopic melanocyte accumulation during embryogenesis. The coexistence of blue nevus and acquired dermal melanocytosis on the back is rare, highlighting the broad spectrum of dermal melanocytosis and the variability of its clinical manifestations. Recognition of such unusual presentations is critical for appropriate diagnosis and management.

## Introduction

Dermal melanocytosis (DM) or benign dermal melanocytic proliferation is a broad group of congenital and acquired pigmentary disorders characterized by the presence of dermal dendritic melanocytes [1]. These conditions are attributed to the arrest of melanocyte migration from the neural crest to the epidermis during embryogenesis. Clinically, DMs can be divided into two main categories: blue nevus (BN), which primarily presents as papulonodular lesions, and other forms of DMs, which typically manifest as patches.

Based on clinical and histopathological differences, BN is commonly classified into two types: common and cellular. Other types of DMs are mainly classified according to their anatomical location: Mongolian spot (lumbosacral region), nevus of Ota (trigeminal nerve area), and nevus of Ito (acromioclavicular region). In addition to the aforementioned classic morphological variants, rare acquired adult-onset cases, known as acquired DM (ADM), are considered separate entities [2].

Herein, we present a case of BN associated with ADM on the back.

## Case presentation

A 46-year-old Japanese woman presented with a black nodule on a blue-greyish hyperpigmented area on her upper back, which had appeared 10 years earlier. The pigmentation was asymptomatic and had appeared without any preceding inflammation, trauma, or sun exposure. The patient was otherwise healthy, did not take any medications, and had no family history of abnormal cutaneous pigmentation.

**Figure 1:**
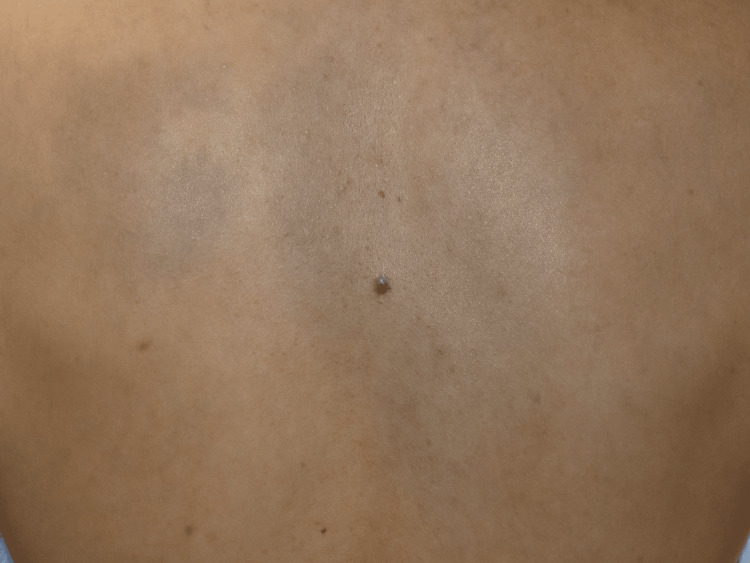
Clinical presentation A 6 × 6 mm blue-black, dome-shaped nodule on an irregularly shaped blue-greyish diffuse patch on the upper back.

**Figure 2:**
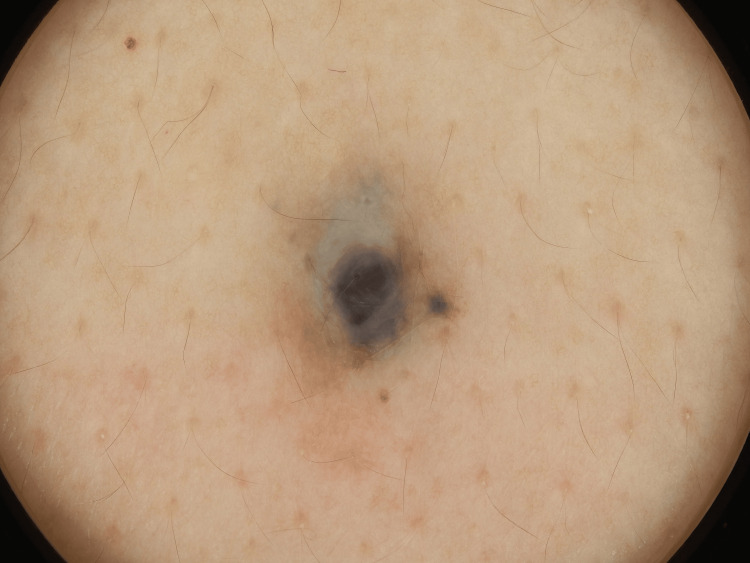
Dermoscopy of the black nodule A homogeneous blue-black, structureless lesion accompanied by a small satellite globule located at the 3 o'clock position.

Physical examination revealed a 6 × 6 mm blue-black, dome-shaped nodule on an irregularly shaped blue-greyish diffuse patch on the upper back (Figure 1). A dermoscopy of the black nodule revealed a homogeneous blue-black, structureless lesion (Figure 2). The lesion was accompanied by a small satellite globule located at the 3 o'clock position.

**Figure 3:**
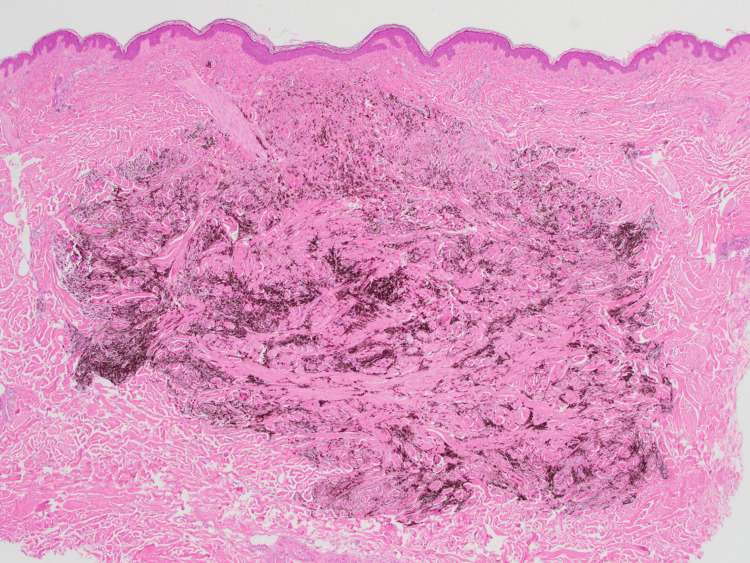
Histopathology of the black nodule An intradermal proliferation of pigmented dendritic melanocytes within a fibrotic stroma (hematoxylin-eosin stain; original magnification ×20).

**Figure 4:**
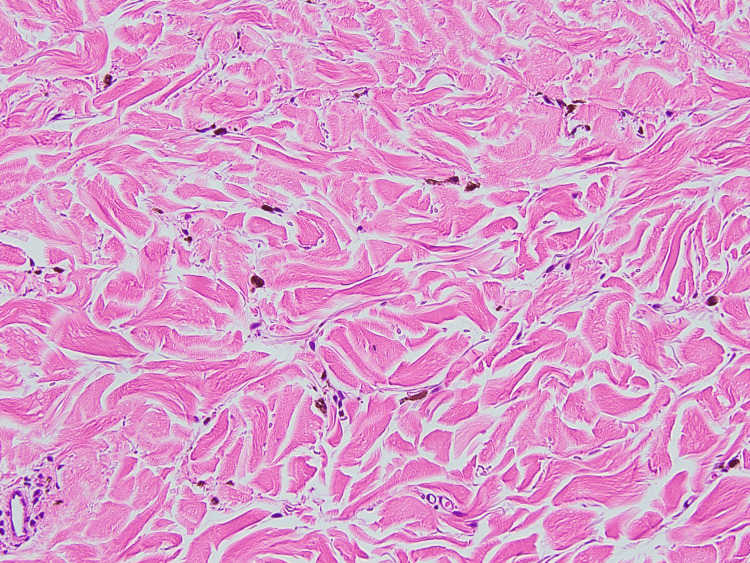
Histopathology of the blue-greyish macule Scattered spindle cells containing melanin among collagen bundles in the dermis (hematoxylin-eosin stain; original magnification ×200).

**Figure 5:**
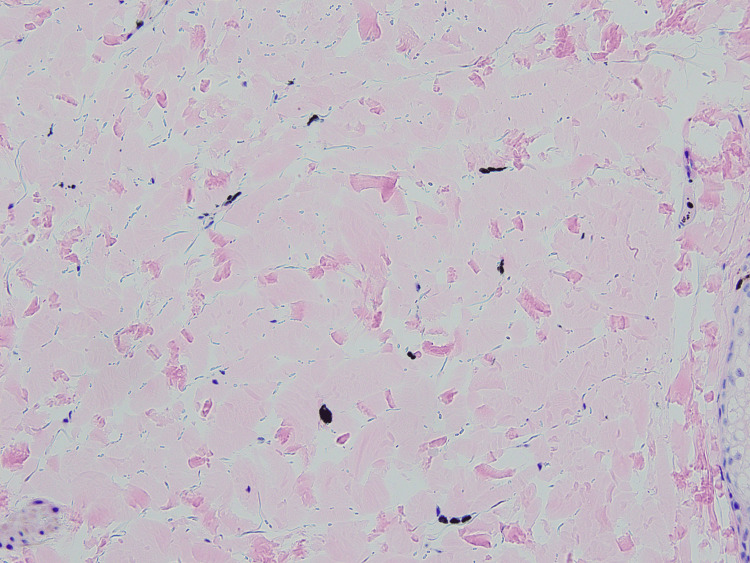
Immunohistochemical analysis The pigmented cells were positive for S-100 stain (original magnification ×200).

Histopathological examination of the black nodule revealed intradermal proliferation of pigmented dendritic melanocytes within the fibrotic stroma (Figure 3). There were no features suggestive of malignancy, such as cytological or nuclear atypia, or mitotic activity. These findings were consistent with the diagnosis of common BN. Furthermore, histopathological examination of the blue-greyish macule revealed normal epidermis and scattered spindle cells containing melanin among the collagen bundles in the dermis (Figure 4). Immunohistochemical analysis revealed that the pigmented cells were positive for S-100 stain (Figure 5). These findings were consistent with the diagnosis of ADM. Our patient declined laser therapy for ADM; therefore, we continued to monitor her condition.

## Discussion

ADM is a heterogeneous group of conditions clinically characterized by grayish-blue macules that typically manifest late in adolescence or adulthood [2]. Several types of ADM have been reported, including acquired bilateral nevus of Ota-like macules (Hori nevus), acquired unilateral nevus of Ota, and extrafacial ADM on the trunk and extremities [3]. The distinction between these entities is primarily based on their different locations, as they are histologically indistinguishable. Histopathology reveals dermal melanocytes, which are bipolar or oval in shape and scattered in the dermis. Although ADM located in nonfacial areas is rare, previous reports have documented cases of ADM on the back, similar to the case presented here [4-7].

The pathophysiology of ADM is not well understood. The higher incidence observed in Asian populations and the observation of familial cases suggest a role for genetic factors. According to the most widely accepted theory, ADM can be attributed to the reactivation of preexisting immature dermal melanocytes [8]. Dormant dermal melanocytes may be present in the dermis from birth due to improper migration from the neural crest to the epidermis during embryonic development. It has been hypothesized that the melanin synthesis pathway may be reactivated by locally produced factors later in life. However, the specific signals that activate the melanin production pathway in these cells remain unknown. Several reactivation triggers have been proposed, including solar radiation, local inflammation, trauma, and medications. In some cases, such as the present one, no clear triggering factors can be identified. Our patient refused laser therapy; however, it is currently regarded as the most effective treatment for ADM if needed [9].

BN most commonly presents in children and young adults as either a congenital or acquired papulonodular lesion [10]. BN is sub-classified into common and cellular types according to its clinical and histological characteristics. Common BN typically presents as solitary, uniformly blue to blue-black, dome-shaped papules less than 10 mm in size. Histologically, they are composed of loose intradermal fascicules of spindle and dendritic melanocytes in a fibrous background. In contrast, cellular BN tends to be larger, with a more elevated nodule or plaque measuring at least 10 mm in diameter. Histologically, cellular BN is characterized by a biphasic pattern of densely pigmented dendritic cells and distinct areas of spindle-shaped melanocytes with relatively pale cytoplasm and scant melanin [11]. Clinically, BN is diagnosed using dermoscopy and is typically described as a homogeneous, structureless lesion, though it may not always appear bluish [12].

Pathophysiologically, BN is hypothesized to develop due to the ectopic accumulation of melanocytes retained in the dermis during their migration from the neural crest to the epidermis. Consequently, BN and its pathophysiology appear to share common features with ADM, and both are classified under the DM category [1]. However, the coexistence of BN and ADM, as presented here, is uncommon and, to the best of our knowledge, has not been previously reported. This case illustrates the diverse clinical presentations of DM. Another notable feature in our case was the presence of a satellite lesion adjacent to the main BN lesion. Melanocytic nevi with satellite lesions are usually indicative of a locally advanced malignant melanoma. However, in very few cases, including ours, BN may be accompanied by satellite lesions with several blue globules adjacent to the main lesion [13]. It has been reported that satellite lesions may spread through the vascular and lymphatic systems surrounding the BN [14]. In this case, BN exhibited entirely benign cytological characteristics, indicating that regular follow-up was the appropriate course of action.

## Conclusions

Herein, we present a case of an unusual acquired pigmentary disorder, BN, associated with ADM on the back. This case highlights the broad spectrum of DM and its highly variable clinical manifestations. To the best of our knowledge, no prior reports have described BN with ADM, suggesting that it may be under-recognized. Although rare, awareness of this condition is crucial for accurate diagnosis and management.
